# Effects of NO_2_ and Ozone on Pollen Allergenicity

**DOI:** 10.3389/fpls.2016.00091

**Published:** 2016-02-04

**Authors:** Ulrike Frank, Dieter Ernst

**Affiliations:** Institute of Biochemical Plant Pathology, Helmholtz Zentrum München – German Research Center for Environmental HealthNeuherberg, Germany

**Keywords:** pollen, allergens, NO_2_, ozone, air pollution

## Abstract

This mini-review summarizes the available data of the air pollutants NO_2_ and ozone on allergenic pollen from different plant species, focusing on potentially allergenic components of the pollen, such as allergen content, protein release, IgE-binding, or protein modification. Various *in vivo* and *in vitro* studies on allergenic pollen are shown and discussed.

## Introduction

Allergic diseases are increasing in Europe. The potential reasons discussed for this trend include climate change and anthropogenic air pollution (Kramer et al., 2000; Ring et al., 2001). It has been shown that ozone and NO_2_, which are two important air pollutants, can have an adverse effect on human health, e.g., the lung function, such as the initiation of lung inflammation by ozone (Kagawa, 1985; Uysal and Schapira, 2003). These air pollutants not only affect human health but also impact plants and their pollen. Ground level ozone, which is a secondary pollutant, is the result of a photochemical reaction of volatile organic compounds and nitrogen oxides (NO_x_), which are mainly produced by combustion processes during energy production, industrial processing or car traffic. Ozone again can also interact with NO_x_. In rural areas, NO_2_ concentrations of up to 20 ppb can be measured, whereas in urban traffic regions, values up to 90 ppb are detectable. In case of ozone, ozone levels along high traffic roads are often low, since ozone is depleted due to the high NO-concentration. At rural sites ozone concentrations often are higher, even so less precursors components are available, since polluted air is transported to the country sites and can react to ozone while the transport. The information threshold for ozone is 1-h average of 90 ppb, but on sunny days alert threshold level of 120 ppb 1-h average can be reached (www.eea.europa.eu).

Air pollution can impact pollen morphology, the pollen cell wall, the pollen protein content or protein release from the pollen as well as the pollen protein itself. In addition, the pollen coat, which is a complex mixture of pigments, waxes, lipids, aromatics and proteins (Edlund et al., 2004), might be impaired due to air pollution. Regarding pollen allergenicity, not only allergenic proteins do play a role, but pollen-derived lipids, which are also called pollen-derived lipid mediators (PALMs), also interact with the immune system and can modify the allergenic reaction (Traidl-Hoffmann et al., 2003; Bashir et al., 2013).

## The Impact of Ozone on Pollen Allergenicity

Concerning the effect of traffic-/industrial-related air pollution on allergenic pollen, most studies have been interested in the allergens, including the allergen content, the possibility of allergen/protein release from the pollen or possible modifications to the allergens. Interestingly, approximately one-fourth of known plant allergens are pathogen-related proteins (PR-proteins), which are induced by different biotic and abiotic stresses, including ground-level ozone (Sandermann et al., 1998; Hoffmann-Sommergruber, 2002). One example is Bet v 1, the major allergen from birch (*Betula pendula*), which is a PR-10 protein. Beck et al. (2013) showed that the Bet v 1 allergen content is positively correlated with increasing ozone levels. This *in vivo* study also indicated significantly larger wheal and flare sizes in patients pricked with pollen extracts from high ozone stands, which was consistent with the higher Bet v 1 content in the pollen (Beck et al., 2013). Another PR protein known as an allergen is the thaumatin-like protein Cup a 3 from *Cupressus arizonica*, which was shown to increase under polluted air conditions (Cortegano et al., 2004; Suárez-Cervera et al., 2008). A further study on another tree species, *Pinus radita*, showed greater allergenicity in terms of prick test and specific IgE binding in connection with higher ozone levels in unpolluted areas (García-Gallardo et al., 2013). The sensitivity to ozone seems to be both species- and concentration-dependent. In an *in vitro* study with ozone concentrations of half, equal and four times the standard limit according to the European Union Directive 2008/50/EC on ambient air quality, the pollen of *Acer negundo*, *Platanus* ssp. and *Quercus robur* showed differences in the total soluble protein (TSP) content. Whereas *Q. robur* and *Platanus* ssp. exhibited significantly reduced protein content under all treatments, *A. negundo* only showed a decrease when it was fumigated with the highest ozone concentration (Ribeiro et al., 2013). Concerning the specific IgE reactivity to *Q. robur* and *A. negundo* pollen extracts, the majority of the tested sera showed increased or unchanged IgE activity compared to the control, but for *Platanus* ssp., the untreated samples showed the highest IgE binding (Ribeiro et al., 2014). Increased allergen contents due to elevated ozone have also been shown for other plant species, such as *Lollium perenne* and *Secale cereale* (Masuch et al., 1997; Eckl-Dorna et al., 2010). A study on grass pollen (*Phleum pratense*) fumigated *in vitro* with 100 ppb ozone for 4 hours resulted in the acidification of several allergens (Phl p 1 b, 4, 5, and 6) as well as decreased IgE recognition of the allergens Phl p 1, 2, 6, and 13 in immunoblots, as explained by the mechanical loss of allergens from altered pollen grains (Rogerieux et al., 2007; **Table 1**). The exposure of *Phleum pratense* pollen to increasing ozone-concentrations from 100 ppb up to 5 ppm resulted in a significant increase in the naturally released pollen cytoplasmic granules (PCG), which are also known to contain allergens, and in more damage to the pollen grain. This mechanism of allergen release might explain the increase in thunderstorm asthma (Motta et al., 2006). Two other studies on *in vivo* and *in vitro* fumigated ragweed pollen did not find any differences in the allergen content of the major allergen Amb a 1 (Pasqualini et al., 2011; Kanter et al., 2013), but differences in the pollen cell wall and increased NADPH oxidase activity could be detected. NADPH oxidase activity was already shown to influence allergenic reactions due to the release of reactive oxygen species (Bacsi et al., 2005). In addition, cell wall modifications might affect immune reaction: in ozone-fumigated ragweed pollen, reduced levels of wax compounds have been detected, and high ozone levels resulted in an altered lipid composition of birch pollen, which led to a modulated immune response (Beck et al., 2013; Kanter et al., 2013).

**Table 1:** Studies of the effect of NO_2_ and/or ozone on the allergenic potential of different pollen species.

Species	Pollutant	Concentration	Exposure time	Allergen	IgE binding	Reference
*Acer negundo*	O_3_	30–235 ppb	6 h		↑	Ribeiro et al., 2014
	NO_2_	150–300 ppb	6 h		↑	Sousa et al., 2012
*Ambrosia artemisiifolia*	O_3_	80 ppb	Growing season	Amb a 1=		Kanter et al., 2013
	O_3_	100 ppb	7 day	Amb a 1=		Pasqualini et al., 2011
	NO_2_	80 ppb	Growing season	Amb a 1 ↑	↑	Zhao et al., 2015
	NO_2_	High traffic roads			↑	Ghiani et al., 2012
*Betula* ssp.	O_3_	Outside stands		Bet v 1 ↑		Beck et al., 2013
*Betula pendula*	NO_2_	34/67 ppb	6/48 h		↑	Cuinica et al., 2014
*Carpinus betulus*	NO_2_	34/67 ppb	6/48 h		↑	Cuinica et al., 2014
*Lolium perenne*	O_3_	65 ppb outside stands	2 weeks –	Phl p 5 ↑ Phl p 5 ↑		Masuch et al., 1997
*Phleum pratense*	O_3_ NO_2_ O_3_/NO_2_	100 ppb 2000 ppb 100 ppb/2000 ppb	4 h		↓ ↓ ↓	Rogerieux et al., 2007
*Pinus radita*	O_3_	Outside stands			↑	García-Gallardo et al., 2013
*Secale cereale*	O_3_	80 ppb	107 days	Phl p 1 ↑ Phl p 5 ↑ Phl p 6 ↑ Profilin ↑		Eckl-Dorna et al., 2010

*↑ = increased; ↓ = reduced*.

## NO_2_ is Impacting the Pollen Allergenicity

Several studies have been performed examining the influence of NO_2_ on pollen allergenicity. Three *in vitro* studies exposing grass pollen (*P. pratense*) to artificially high NO_2_-concentrations from 500 ppb up to 5000 ppm resulted in a dose-dependent increase in PCGs and a dose-dependent increase in pollen grain damage (Motta et al., 2006) as well as a reduction of IgE binding to Phl p 2, 5b, and 6 (Rogerieux et al., 2007). In addition, a direct correlation of NO_2_ uptake by the pollen with a higher T_H_2 response of human cells could be demonstrated (Chassard et al., 2015), which indicated that NO_2_ can interact with the pollen grains and thus leads to degradation of pollen structure and changes in protein content. Other studies on ragweed (*Ambrosia artemisiifolia*) showed higher allergen levels and elevated IgE binding under elevated NO_2_ concentrations, where the higher IgE recognition was mainly due to the major allergen Amb a 1 (Ghiani et al., 2012; Zhao et al., 2015). Zhao et al. (2015) also showed IgE binding to a new allergen in ragweed with homology to Hev b 9 from the rubber tree, induced by elevated NO_2_ (Zhao et al., 2015). However, contrary results were found for several tree pollen species fumigated with NO_2_ concentrations between 34 and 300 ppb. *Betula pendula* and *Carpinus betulus* showed decreased pollen viability when exposed to NO_2_ and had a lower TSP content. *Ostrya carpinifolia* also showed a lower TSP compared with the control. This study also demonstrated increased pollen allergenicity for *C. betulus*, *O. carpinifolia*, and *B. pendula* when exposed for a short time to relatively small NO_2_ concentrations (Cuinica et al., 2014). *A. negundo* also showed similarly elevated IgE binding but slightly increased TSP, whereas lower TSP was detected for *Ricinus communis* (Bist et al., 2004; Sousa et al., 2012).

Air pollution due to NO_2_ can result in post-translational modification such as S-nitrosylation or the nitration of pollen proteins. Zhao et al. (2015) showed that the fumigation of ragweed plants with elevated NO_2_ concentrations throughout a growing season resulted in increased overall S-nitrosylation, and LC-MS/MS analysis of the S-nitrosylated proteins indicated the major ragweed allergen Amb a 1 as a possible candidate for S-nitrosylation (Zhao et al., 2015). Another important aspect is the nitration of allergens in pollen, which can be caused by the presence of ozone and NO_2_. Studies on aerosolized proteins showed an ozone dependent increase of nitration due to NO_2_ (Shiraiwa et al., 2012). Franze et al. (2005) showed that the major allergen from birch Bet v 1 is effectively nitrated in the presence of NO_2_ and ozone but that the nitration degree was substantially lower when the proteins were exposed to NO_2_ alone, thus indicating that reactive species formed upon the interaction of ozone and NO_2_ play a major role in the nitration. This nitration affects the allergenic potential of the birch pollen. The nitration of Bet v1 results in stronger proliferation of Bet v 1-specific T cell lines, and IgE binding to nitrated Bet v 1 is higher than IgE binding to Bet v 1 (Gruijthuijsen et al., 2006; Karle et al., 2012). An oligomerization of Bet v 1 due to nitration was also observed, which resulted in lower sensitivity to endolysomal degradation (Ackaert et al., 2014). This posttranslational modification of allergens provides a rationale for the increase in allergic diseases in air polluted regions.

Summarizing the different studies, little is yet known about the molecular mechanisms of the effects of ozone and NO_2_ on pollen, and more research is needed on that point. However, the existing research already clearly indicates dose-dependent and species-specific impacts of these air pollutants, which in most cases result in heightened allergenicity.

## Author Contributions

UF and DE contributed equally to the manuscript concerning writing and conception.

## Conflict of Interest Statement

The authors declare that the research was conducted in the absence of any commercial or financial relationships that could be construed as a potential conflict of interest.
